# Association between social capital and depression among older people: evidence from Anhui Province, China

**DOI:** 10.1186/s12889-020-09657-7

**Published:** 2020-10-16

**Authors:** Zhongliang Bai, Zhiwei Xu, Xiaoru Xu, Xia Qin, Wenbiao Hu, Zhi Hu

**Affiliations:** 1 Department of Epidemiology and Biostatistics, School of Public Health, Anhui Medical University, Hefei, 230032 China; 2 Department of Health Services Management, School of Health Services Management, Anhui Medical University, Hefei, 230032 China; 3 School of Public Health and Social Work, Institute of Health & Biomedical Innovation, Queensland University of Technology, Brisbane, 4059 Australia; 4 School of Public Health, Faculty of Medicine, University of Queensland, Brisbane, 4059 Australia

**Keywords:** Social capital, Depression, Elderly, Mental health, China

## Abstract

**Background:**

To examine the relationship between social capital and depression among community-dwelling older adults in Anhui Province, China.

**Methods:**

A cross-sectional study was conducted among older people selected from three cities of Anhui Province, China using a multi-stage stratified cluster random sampling method. Data were collected through questionnaire interviews and information on demographic characteristics, social capital, and depression was collected. The generalized linear model and classification and regression tree model were employed to assess the association between social capital and depression.

**Results:**

Totally, 1810 older people aged ≥60 years were included in the final analysis. Overall, all of the social capital dimensions were positively associated with depression: social participation (coefficient: 0.35, 95% CI: 0.22–0.48), social support (coefficient:0.18, 95% CI:0.07–0.28), social connection (coefficient: 0.76, 95% CI:0.53–1.00), trust (coefficient:0.62, 95% CI:0.33–0.92), cohesion (coefficient:0.31, 95% CI:0.17–0.44) and reciprocity (coefficient:0.30, 95% CI:0.11–0.48), which suggested that older people with higher social capital had a smaller chance to develop depression. A complex joint effect of certain social capital dimensions on depression was also observed. The association with depression and the combinative effect of social capital varied among older adults across the cities.

**Conclusions:**

Our study suggests that improving social capital could aid in the prevention of depression among older adults.

## Background

The proportion of older people (≥ 60 years) in many countries is increasing [1]. China has the highest number of older people in the world with a rapidly aging population [2], and geriatric depression remains a great public health challenge [3]. Recent evidence has suggested approximately one-fifth of older adults in China have depressive disorders [4]. Depression cannot only impair functional ability, reduce the quality of life and increase the mortality of older adults, but also inflicts a heavy economic burden upon older adults themselves, the society, and the healthcare system [5].

One of the key measures of preventing depression in older people is to identify associated risk factors of depression. Common risk factors of depression include advanced age, female, unfavorable economic levels [5, 6]. In recent years, increasing studies revealed that older adults are prone to depression if there is an alteration in social roles, social and family settings, and adverse life events (i.e. diseases or loss of spouse) [7]. With the development of social determinants of health, the role of social capital in the mental health of human beings has been increasingly recognized [8]. Social capital is a multi-faced concept and includes multiple dimensions, each of which is used to describe a phenomenon pertaining to social relations at the individual and societal levels [6, 9]. Studies in many countries including China [6, 10–12] have shown that social capital is associated with depression in older people and increasing attention has been focused on the effect of social capital on geriatric mental health [13].

However, previous studies looking at the association of social capital with depression among older people used various dimensions to measure social capital and reported inconsistent results. For instance, a study in Korea used trust and reciprocity to measure social capital and suggested that low trust and reciprocity levels were associated with depressive symptoms in older people [6]. Similarly, another study in Korea explored the relationship between social capital (measured with network and trust) and depression among urban older adults and found that trust in social capital was associated with depression, while network was not [14]. A study in China surveyed the association of social capital (assessed with trust, reciprocity, social network, and social participation) with depression among urban older people, and revealed that trust, reciprocity, and social network were significantly associated with depression while social participation was not [11]. Therefore, understanding and comparing the association between social capital depression among older adults across regions requires a standard or highly accepted and validated method to measure social capital.

Although a number of risk factors such as age, female, unfavorable economic levels and social capital, have been investigated in previous research [5–7, 11], it remains unclear if there is a synergistic or antagonistic effect between social capital and other risk factors on depression. In practice, older adults are exposed to two or more risk factors like low economic level and low trust and reciprocity, making them more vulnerable to depression [6]. It is thus needed to investigate how risk factors interact to influence the depression in later life. Knowledge about these findings will yield more tailored and accurate measures to protect older people from depressive disorders.

Therefore, in the current study, we aimed to examine the association of social capital with depression in older adults in China. Specifically, we first examined whether six social capital dimensions in this study were associated with depression and then further explored the joint effect of social capital and other common risk factors on depression.

## Methods

### Study population and data collection

**Fig. 1:**
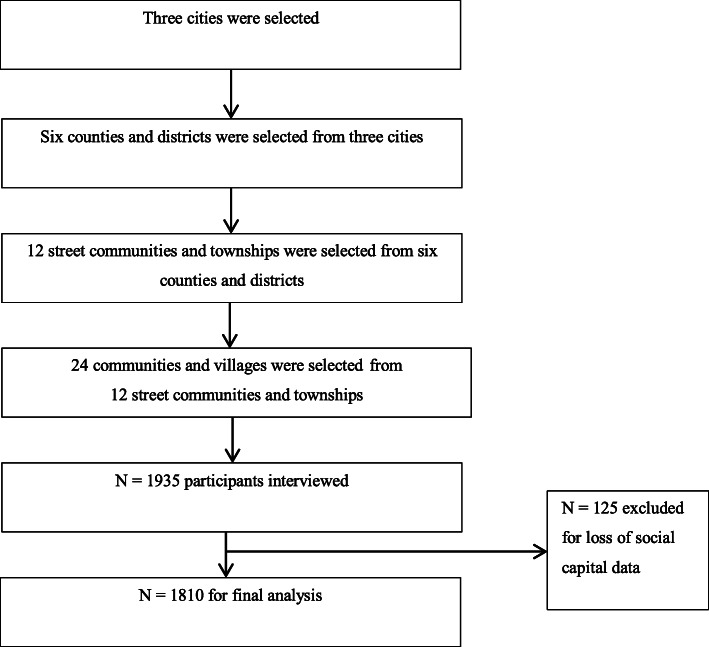
The flowchart of the sampling process

According to the statistics of Anhui Statistics Bureau in 2016 [15], the gross domestic product (GDP) per capita was CNY (Chinese yuan, 1 US $ equals about CNY 7.10) 80,138 for Hefei, CNY 40,740 for Xuancheng, and CNY 17,642 for Fuyang. In addition, among sixteen cities of Anhui Province, Hefei, Xuancheng, and Fuyang ranked first, eighth, and last, respectively, in GDP per capita. These three cities were selected in this study. Based on the geographic location and economic levels [15], a multi-stage stratified cluster random sampling method was employed to recruit participants in order to have a representative sample. At the first stage, we selected three prefecture-level cities from the sixteen prefecture-level cities in Anhui province, China: Fuyang (north, lower economic level); Xuancheng (south, middle economic level); Hefei (central, higher economic level, the capital city of Anhui). Then, in each prefecture-level city, one county and one district were selected randomly. A total of six counties and districts were selected in this study. Next, in each selected county and district, one street community and one township were randomly selected and a total of 12 street communities and townships were used as sample sites for this study. Lastly, in each selected street community and township, two communities and two villages were selected randomly and 24 sampling areas were ascertained (Fig. 1). More information about our sampling process can also be founded elsewhere [16].

Between July and September 2017, we conducted cross-sectional surveys in these 24 sampling areas. Based on the local household registry, individuals aged ≥ 60 years were determined. Aided by local community workers, skilled and trained graduate students from Anhui Medical University visited each participant and conducted face to face interviews using a structured questionnaire. The participants received a verbal description of the purposes and procedures of the study and informed consent is needed before the interview. The process of data collection took about 40 min. Each respondent was compensated with a gift of about 2 US dollars (CNY 15) for the time and cooperation after the interview. Individuals who were not able to carry out proper verbal communication, due to being deaf or mute and dementia or cognitive impairments, were excluded. In the present study, 1935 older adults were interviewed, of which 1810 (93.54%) were eligible for analysis, with 567 in Fuyang, 603 in Xuancheng, and 640 in Hefei, respectively.

### Measurement of social capital

Based on the framework of the World Bank’s Social Capital Assessment Tool and previous works of our research group [9, 16–18], six dimensions of social capital were included in the present study: social participation, social support, social connection, trust, cohesion, and reciprocity. We selected 22 commonly used and easily understandable items to measure social capital and adapted them to the Chinese context. In the present study, the five-point Likert scale was adopted in the social capital questionnaire, and respondents were asked to rate their agreement (1“never”, 2 “seldom”, 3 “usually”, 4 “often”, and 5 “more often”). The measurement of social capital has also been described elsewhere [16] and more detailed information about the questionnaire can be found in the supplementary file (Additional file 1).

For each domain of social capital, answers to the items were summated to obtain an overall score with higher scores indicating better social capital status. Construct validity was tested to estimate the validity of our instrument by exploring the correlations of each item of social capital dimension and the scores of social capital dimensions, respectively, where large effect size (correlation coefficient ≥ 0.50) was observed in all magnitude [19], indicating construct validity of our instrument. Internal consistency was calculated to prove the reliability of the measurement tool. Cronbach’s α of the questionnaire was 0.919, showing an excellent internal consistency for our scale with this specific sample. For each dimension, Cronbach’s α for social participation, social support, social connection, trust, cohesion, and reciprocity was 0.752, 0.921, 0.767, 0.883, 0.940, and 0.869, respectively.

### Measurement of depressive symptoms

The Zung Self-Rating Depression Scale (SDS), a widely used screening tool for depressive feelings [20, 21], was adopted to assess the depression status of the participants. Construct validity was tested to estimate the validity of our instrument by exploring the correlations of each item of the scale and the scores of depression. The correlation coefficient ranges from 0.682 to 0.888, which shows a large effect size (correlation coefficient ≥ 0.50) [19], indicating a good validity of our instrument. Cronbach’s α of the scale was 0.965, indicating excellent internal consistency in this sample.

In this study, we selected 16 items to measure depression of older adults, which were more understandable and acceptable for the Chinese community older dwellers. We summated the items to calculate the total depression score, ranging from 16 to 64, with higher scores indicating a lower likelihood of depression.

### Assessment of demographic variables

Information on the demographic and health-related variables was collected. These variables included age (60–64, 65–69, 70–74, ≥ 75, years), gender (male, female), body mass index (BMI, kg/m^2^), residence (urban, rural), living status (living alone, living with spouse/children/grandchildren and else), marital status (married/cohabited, never married/divorced, widowed), and education (primary school and below, junior high school, high school, college and above). Information on smoking and drinking status was also collected.

### Statistical analysis

Continuous variables were presented as mean ± standard deviation and range, categorical variables were presented as number (%). A general linear model (GLM) was used to initially investigate the relationship between different social capital dimensions and depression scores. The GLM model can be specified as follows:

$$ Depression\ scores\approx \alpha +{\beta}_1 Social\ capital\ dimensions+{\beta}_2{Confounders}_1+\dots +{\beta}_n{Confounders}_n $$

where depression score is the dependent variable; *α* is the intercept; social capital dimensions refer to the above-mentioned six dimensions of social capital and *β*_1_ is the corresponding coefficient; *β*_2_*Confounders*_1_ + … + *β*_*n*_*Confounders*_*n*_ indicate potential confounders in the model and their corresponding coefficients were *β*_2_…*β*_*n*_. In this model, we considered age, gender, body mass index, residence, living status, marriage status, education, smoking, and drinking status as potential confounders as previous studies have shown that these confounders are associated with depression in later life [4, 5, 22, 23]. Other confounders such as shorter sleeping time and physical disability [23] were not included as no data was available for this study. Collinearity between all independent variables was not existing according to the variance inflation factor (VIF) results (Additional file 2). At last, to explore the combinative relationship between social capital and depression, a classification and regression tree (CART) model was developed by dividing all social capital dimensions (social participation, social support, social connection, trust, cohesion, and reciprocity) and demographic variables into subsets. The classification and regression tree (CART) model is a flexible, robust, and non-parametric model that was previously used in depression disease study [24, 25]. The best model is defined as having the smallest tree size and an estimated error rate within one standard error of the minimum [26]. The GLM model and CART model were stratified according to the economic levels separately. All statistical analyses were performed with SPSS statistics software, version 23 (SPSS Inc.; Chicago, IL, USA) and R version 3.4.0. *P* < 0.05 was considered statistically significant.

## Results

### Results of descriptive analysis

They included 770 males (42.5%) and 1040 females (57.5%) and their mean age was 71.20 ± 7.51 years (range 60 to 96). Rural residents accounted for 55.7% of the population and 71.66% of the participants did not live alone. In addition, 77.5% of respondents were married or cohabited, 71.3% of participants attended primary school and below, and the majority of respondents were non-smokers (78.0%) and non-drinkers (82.0%). (Table. 1).

**Table 1:** Descriptive analysis results of participants characteristics (*N* = 1810)

Variables	Total N = 1810 ^*^	Fuyang ***N*** = 567 ^*^	Xuancheng ***N*** = 603 ^*^	Hefei ***N*** = 640 ^*^
**Age (years), range (60–96)**	70.00 ± 7.51	70.47 ± 7.55	70.65 ± 7.24	72.37 ± 7.60
60–64	399 (22.0)	146 (25.7)	139 (23.1)	114 (17.8)
65–69	424 (23.4)	138 (24.3)	154 (25.5)	132 (20.6)
70–74	421 (23.3)	119 (21.0)	143 (23.7)	159 (24.8)
≥75	566 (31.3)	164 (28.9)	167 (27.7)	235 (36.7)
**Gender**				
Male	770 (42.5)	220 (38.8)	277 (45.9)	273 (42.7)
Female	1040 (57.5)	347 (61.2)	326 (54.1)	367 (57.3)
**BMI (kg/m**^**2**^**), range (14.5–45.9)**	22.72 ± 3.44	23.48 ± 3.65	22.13 ± 3.30	22.59 ± 3.27
**Residence**				
Urban	801 (44.3)	249 (43.9)	218 (36.2)	334 (52.2)
Rural	1009 (55.7)	318 (56.1)	385 (63.8)	306 (47.8)
**Living status**				
Living alone	243 (13.4)	66 (11.6)	72 (11.9)	105 (16.4)
Living with others	1567 (86.6)	501 (88.4)	531 (88.1)	535 (83.6)
**Marital status**				
Married / cohabited	1402 (77.5)	440 (77.6)	475 (78.8)	487 (76.1)
Never married / divorced	19 (1.0)	4 (0.7)	8 (1.3)	7 (1.1)
Widowed	389 (21.5)	123 (21.7)	120 (19.9)	146 (22.8)
**Education**				
Primary school and below	1291 (71.3)	439 (77.4)	463 (76.8)	389 (60.8)
Junior high school	291 (16.1)	72 (12.7)	75 (12.4)	144 (22.5)
High school	163 (9.0)	45 (7.9)	48 (8.0)	70 (10.9)
College and above	65 (3.6)	11 (1.9)	17 (2.8)	37 (5.7)
**Smoking status**				
Smoking-quitter	99 (5.5)	29 (5.1)	40 (6.6)	30 (4.7)
Smoker	299 (16.5)	93 (16.4)	130 (21.6)	76 (11.9)
Non-smoker	1412 (78.0)	445 (78.5)	433 (71.8)	534 (83.4)
**Drinking status**				
Drinking-quitter	70 (3.9)	33 (5.8)	15 (2.5)	22 (3.4)
Drinker	256 (14.1)	68 (12.0)	102 (16.9)	86 (13.4)
Non-drinker	1484 (82.0)	466 (82.2)	486 (80.6)	532 (83.1)
**Social participation, range (4–20)**	7.34 ± 3.80	5.95 ± 2.67	6.94 ± 3.36	8.95 ± 4.40
**Social support, range (4–20)**	12.06 ± 5.29	11.91 ± 5.46	12.04 ± 4.71	12.20 ± 5.64
**Social connection, range (3–15)**	12.20 ± 2.49	12.23 ± 2.29	12.10 ± 2.70	12.27 ± 2.47
**Trust, range (3–15)**	12.89 ± 2.30	12.96 ± 2.16	12.54 ± 2.48	13.15 ± 2.20
**Cohesion, range (5–25)**	19.36 ± 4.73	19.89 ± 4.08	18.12 ± 5.19	20.06 ± 4.59
**Reciprocity, range (3–15)**	10.54 ± 3.36	10.28 ± 3.53	10.43 ± 3.42	10.88 ± 3.12
**Depression, range (16–64)**	49.13 ± 11.30	46.13 ± 12.54	49.03 ± 12.54	51.87 ± 9.57

Note: * continuous variables are presented as range and mean ± standard deviation, categorical variables are presented as number (%)

### Results of the GLM model

As shown in Table 2, after controlling for confounders, the effects of six dimensions of social capital became attenuated but were positively associated with depression. Specifically, in the total population, with each social capital dimension increased one score, depression scores increased by 0.35, 0.18, 0.76, 0.62, 0.31, 0.30, respectively. However, in cities of different economic levels, social capital dimensions related to depression were varied. In Fuyang (lower economic level), all dimensions were statistically associated with depression, except for reciprocity. In Xuancheng (middle economic level), social connection, reciprocity, and cohesion were positively associated with depression. Meanwhile, social participation, social support, and trust were not statistically related to depression. In Hefei (higher economic level), social support, social connection, trust, and cohesion were positively associated with depression while social participation and reciprocity were not statistically significant.

**Table 2:** The relationship between social capital and depression using GLM

		Unadjusted			Adjusted		
	**Social capital dimensions**	**B(S.E.)**	**95% CI**	***p*-Value**	**B(S.E.)**	**95% CI**	***p*-Value**
**Overall**	Social participation	0.88 (0.07)	0.75–1.01	< 0.001	0.35 (0.07)	0.22–0.48	< 0.001
	Social support	0.73 (0.05)	0.64–0.83	< 0.001	0.18 (0.05)	0.07–0.28	0.001
	Social connection	1.83 (0.10)	1.64–2.02	< 0.001	0.76 (0.12)	0.53–1.00	< 0.001
	Trust	2.11 (0.10)	1.90–2.31	< 0.001	0.62 (0.15)	0.33–0.92	< 0.001
	Cohesion	1.01 (0.05)	0.91–1.11	< 0.001	0.31 (0.07)	0.17–0.44	< 0.001
	Reciprocity	1.31 (0.07)	1.17–1.46	< 0.001	0.30 (0.09)	0.11–0.48	0.001
**Fuyang**	Social participation	1.15 (0.19)	0.78–1.53	< 0.001	0.36 (0.18)	0.00–0.71	0.048
	Social support	0.88 (0.09)	0.71–1.05	< 0.001	0.26 (0.11)	0.04–0.49	0.022
	Social connection	2.30 (0.21)	1.89–2.71	< 0.001	0.76 (0.27)	0.24–1.29	0.004
	Trust	2.66 (0.22)	2.24–3.09	< 0.001	0.76 (0.33)	0.10–1.41	0.023
	Cohesion	1.52 (0.11)	1.30–1.74	< 0.001	0.63 (0.18)	0.29–0.98	< 0.001
	Reciprocity	1.42 (0.14)	1.15–1.68	< 0.001	0.08 (0.19)	-0.29–0.44	0.688
**Xuancheng**	Social participation	0.75 (0.13)	0.50–1.01	< 0.001	0.26 (0.13)	0.00–0.52	0.051
	Social support	0.61 (0.09)	0.43–0.79	< 0.001	0.09 (0.10)	-0.10–0.28	0.354
	Social connection	1.60 (0.15)	1.30–1.90	< 0.001	0.86 (0.20)	0.47–1.25	< 0.001
	Trust	1.75 (0.17)	1.42–2.08	< 0.001	0.30 (0.25)	-0.19–0.80	0.231
	Cohesion	0.80 (0.08)	0.64–0.96	< 0.001	0.27 (0.11)	0.06–0.48	0.011
	Reciprocity	1.26 (0.12)	1.03–1.50	< 0.001	0.44 (0.16)	0.12–0.76	0.008
**Hefei**	Social participation	0.63 (0.08)	0.47–0.80	< 0.001	0.16 (0.08)	0.00–0.32	0.050
	Social support	0.67 (0.06)	0.55–0.79	< 0.001	0.18 (0.07)	0.04–0.32	0.010
	Social connection	1.73 (0.14)	1.46–2.00	< 0.001	0.65 (0.16)	0.32–0.97	< 0.001
	Trust	2.03 (0.15)	1.73–2.32	< 0.001	0.77 (0.21)	0.37–1.17	< 0.001
	Cohesion	0.99 (0.07)	0.85–1.13	< 0.001	0.43 (0.10)	0.23–0.62	< 0.001
	Reciprocity	1.12 (0.11)	0.90–1.34	< 0.001	0.06 (0.13)	-0.20–0.31	0.675

Note: Adjusted by age, gender, BMI, residence, living status, marital status, education, smoking, and drinking status

B: regression coefficient

S.E.: standard error

95% CI: confidence interval of 95%

### Results of the CART model

**Fig. 2:**
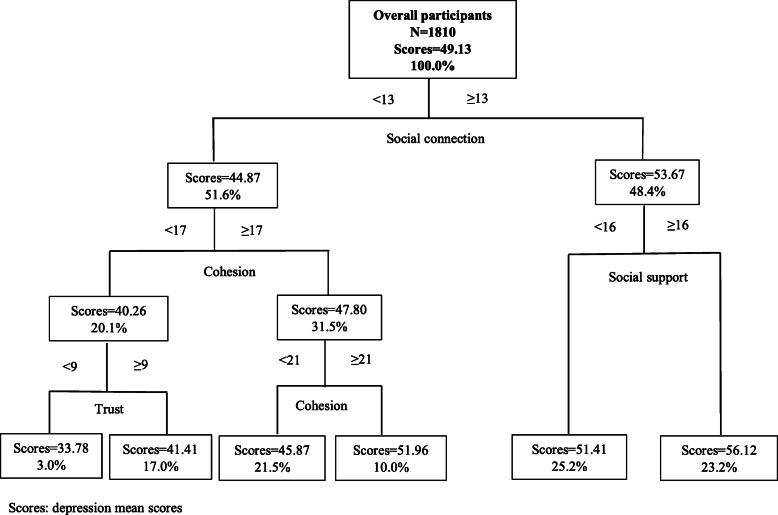
CART results of social capital and depression (*N* = 1810)

**Fig. 3:**
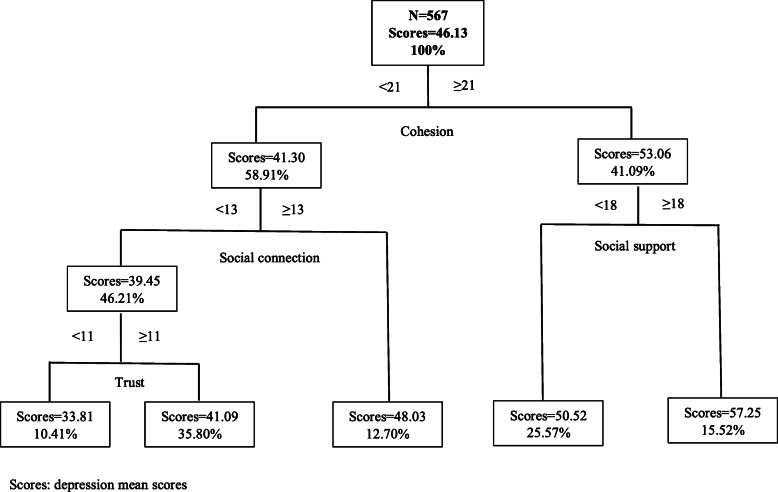
CART results of social capital and depression in Fuyang (N = 567)

**Fig. 4:**
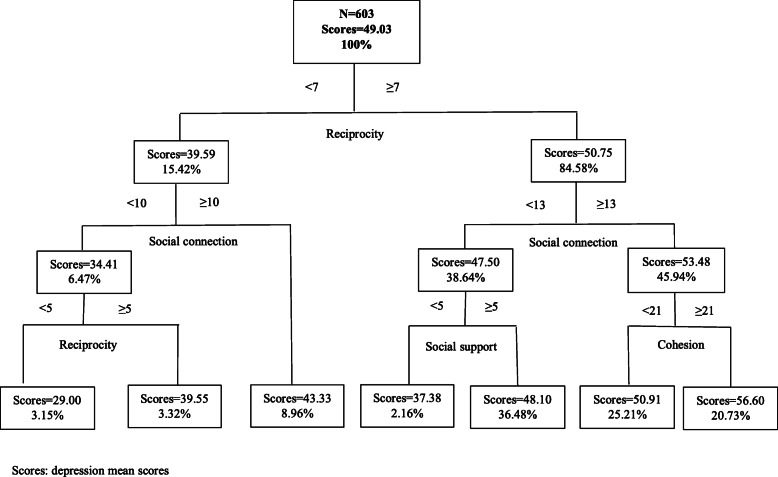
CART results of social capital and depression in Xuancheng (N = 603)

**Fig. 5:**
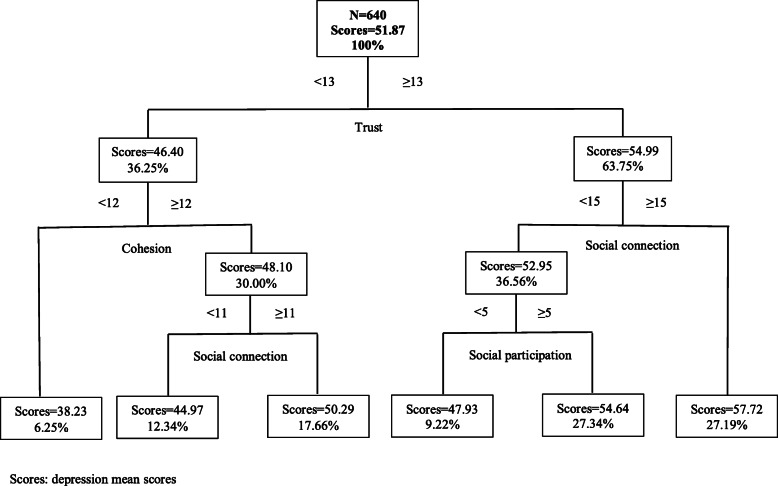
CART results of social capital and depression in Hefei (N = 640)

The joint effect of social capital was observed (Fig. 2). Social connection was the first classifying dimension. Thus, social connection was the most important dimension of social capital and was associated with depression among older people. Overall, older adults whose social connection score was ≥13, and social support score were ≥ 16 showed an increase of 6.99 in the mean depression scores (from 49.13 to 56.12). Such a joint effect of social capital related to depression also varied among older people from areas of different economic levels. (Figs. 3, 4 and 5).

Cohesion was the first classifying factor. Thus, this factor was the most associated with depression among older people in the lower economic level city (Fuyang). Among those participants with higher cohesion score (≥ 21) and social support score (≥ 18), the mean depression scores increased by 11.12 (from 46.13 to 53.06) in comparison to those with lower cohesion and social support scores (Fig. 3).

For older people in the middle economic level area (Xuancheng), reciprocity was the first classifying factor. The results suggested that older adults with higher reciprocity score (≥ 7), social connection score (≥ 13), and cohesion score (≥ 21) were less likely to report geriatric depression, along with an increase of 7.57 in the depression score (from 49.03 to 56.60) (Fig. 4).

Among older people in the higher economic level city (Hefei), trust was the first classifying dimension, which was mostly associated with depression. Participants whose trust score ≥ 13 and social connection score ≥ 15 had higher depression scores (from 51.87 to 57.72) (Fig. 5).

## Discussion

The present study examined the relationship of social capital with depression in later life from a representative sample in Anhui, China. The results revealed the correlation of social capital with geriatric depression and the joint effect of certain social capital dimensions on depressive symptoms. Moreover, such findings persisted when separated by different economic level areas.

We found that a higher level of social capital was associated with a lower likelihood of experiencing depressive symptoms after adjustment for confounders in the total population, which is consistent with previous studies [6, 10, 11, 27]. Li et al. [10] and Han et al. [6] showed that a lower level of social capital (concerning trust and reciprocity) was connected associated with suffering from depression among people in their later life. In line with a prior study [28], we also found social connection could reduce the risk of depression among older adults. Similar to our findings, Haseda et al. [29] suggested that more exchanges in social support were associated with a lower risk of depression, especially among male older adults. We also observed that older people with more cohesion were less likely to have depression, which is consistent with previous findings [28, 30, 31]. However, different from our findings, a previous study found no association between social participation and depression [11]. One possible reason for this inconsistency may be that physical function wellness has been proven to influence the association of social participation with depression among older people [11, 31, 32]. Thus, more study is needed to further examine this conclusion in the future.

We also observed that certain dimensions of social capital matter for depression among older people at different economic level cities. Specifically, social capital in terms of social participation, social support, social connection, trust, and cohesion is statistically correlated with depressive symptoms among older adults from a lower economic level area. Meanwhile, social capital regarding social connection, cohesion, reciprocity is found to be associated with depression among older people from a middle economic level area. Finally, social capital concerning social support, social connection, trust, and cohesion is significant for older people from an area with a higher economic level. To our knowledge, this is the first study to explore the association between social capital and depression among older adults stratified by different economic levels. This mixed pattern of associations reveals that certain dimensions of social capital play a role across the economic level, which further highlights that economic levels play a significant role in constructing and building social capital [33, 34]. The significance of this finding indicates that social capital should be included when taking measures to address the issues of depression among older people and adds to the limited literature on the disparity in social capital in areas of different economic levels. In addition, this underscores the necessity to assess social capital with several dimensions instead of just one single dimension [9].

Another significant finding of this study is the joint effect of certain dimensions of social capital on depression. In the total population, older adults who had greater social participation and social support were less likely to develop depression. Similarly, such a joint effect also varied among older people from areas of different economic levels. That is, older people from an area of a lower economic level who reported greater cohesion and social support were prone to be depression-free. By contrast, older people residing in an area of middle economic level who reported greater reciprocity, social connection, and cohesion were less likely to experience depressive symptoms. Meanwhile, older adults from an area of higher economic level who had greater trust and social connection were less likely to have depression. Similar to our results, a previous study also suggested that older adults living in lower socioeconomic status locations prefer to communicate and interact with neighbors, thus generating higher social cohesion which is good for depression prevention [35]. Besides, in China, poor areas are the core of help measures under the national policy “Poverty Alleviation” in the past decades; as a result, people in these areas can get help and support from various channels [36]. Previous studies also concluded that older individuals with worse reciprocity and social connection had a higher risk of depression [6, 31, 37], but they did not categorize older people with a middle economic level, and more evidence is needed to examine the association between social capital and depression within a region with different economic statuses. Likewise, a study found that older people living in a higher economic community with greater trust with family members were less likely to be depressed [6, 38]. By looking at these findings, we reconfirmed the significant role of social capital in maintaining the mental health of older populations, which is in line with previous studies [6, 11] and broadens our understanding of the role of social capital across areas of different economic levels.

Previous studies have found that economic levels are also of importance for the health of an individual as higher economic levels are associated with better utilization of public resources and facilities and a more sound social welfare system, which confer benefits on less likely to experience depression [33, 39]. Besides, some factors of a region including economic levels have been found to influence the construction and building of social capital [33], and pathways that connect social capital with health may vary in different economic settings [34]. Therefore, when linking social capital to health outcomes, economic levels should be well considered [39].

Some suggestions for preventing the onset of depression in later life can be provided based on our results. First, variations in possession of certain dimensions of social capital by economic levels may have implications for devising programs that aim at preventing depression and suggest that such programs should be compatible with the economic setting of a region. Besides, to maximize the function of social capital in maintaining mental wellness, measures including certain social capital should take the joint effect of social capital into account. For example, more attention should be paid to social connection and social support when devising measures to reduce the risk of being depressed among older people.

However, the study has several limitations. First, this is a cross-sectional study, which could not provide sufficient evidence to establish causality. Second, the subjects of this study were only selected from three cities in Anhui province, and the results may not apply to other regions in China. Third, there is no commonly used measurement tool of social capital, which makes the comparison with other studies difficult. Lastly, another limitation is that the confounders in this study were not comprehensive enough (e.g., economic level, alteration in social roles, social and family settings, adverse life events [7], and physical disability [23]).

Nevertheless, this study has some strengths. First, our study has explored the relationship between social capital and geriatric depression using a representative sample status with a good response rate in Anhui, China. Our results provide important evidence concerning the role of social capital on depression. Second, the study used simple, reliable, valid assessment tools to obtain data about social capital; Also, the CART model found a joint effect of social capital on depression, which implies that a comprehensive and complex analytical method could be used to design more accurate and specific measures to reduce the incidence of depression in later life.

## Conclusions

The present study provides evidence on the relationship between social capital and geriatric depression shows that social capital is associated with depression. Specifically, in areas of lower socioeconomic level, older people with better cohesion and social support results were less likely to have depression. Meanwhile, in the middle economic level areas, individuals who had lower reciprocity and social connection scores were more prone to have depression. In the higher economic level areas, older adults whose trust and social connection scores were higher were more likely to be mentally healthy.

## Supplementary information

**Additional file 1.** Social capital and elderly depression questionnaire (English version), detailed information about the measurement tool.

**Additional file 2.** Results of collinearity analysis.

## Data Availability

The datasets generated during and/or analyzed during the current study are available from the corresponding author on reasonable request.

## Abbreviations

GDP: Gross Domestic Product
CNY: Chinese yuan
SDS: The Zung Self-Rating Depression Scale
BMI: Body Mass Index
GLM: General Linear Model
VIF: Variance Inflation Factor
CART: Classification And Regression Tree
