# Supplementary material for: Association between social capital and depression among older people: evidence from Anhui Province, China
Source: BMC Public Health. 2020 Oct 16;20:1560. doi: 10.1186/s12889-020-09657-7 (PMC7565750; doi:10.1186/s12889-020-09657-7)
Supplement: Supplementary file 1 — Additional file 1. Social capital and elderly depression questionnaire (English version), detailed information about the measurement tool. [file 12889_2020_9657_MOESM1_ESM.docx]

**Social Capital and Elderly Depression Questionnaire**

**(English version)**

**PART A DEMOGRAPHIC INFORMATION**

A1. How old are you years?

A2. What is your gender: 1. Mele 2. Female

A3. What is height: meter

A4. What is your body weight? Kilogram

A5. Where is your residence: 1. Urban 2. Rural

A6. Currently, who do you living with?

1. Living alone 2. Spouse 3. Children 4. Grandchildren 5. Else

A7. Currently, what is your marriage status?

1. Married/cohabited 2. Unmarried 3. Divorced 4. Widowed

A8. What is your education status?

1.primary school and below 2. junior high school 3. high school 4. college and above

A9. Do you smoke? (Smoking means smoking at least one a day for more than half a year)

1. currently quit smoking ;2. Yes, smoking, jump to A9.2; 3 No, do not smoke, jump to A10

A9.1 If you have quit smoking, how long have you quit smoking? (months)

A9.2 How many years have you smoked? (years)

A9.3 How many cigarettes do you have currently/ before you quit smoking?

A10. Do you drink regularly? (Drinking means drinking at least once a week for more than half a year)

1. currently abstaining from alcohol;2. Yes, drink, jump to A10.2; 3 No, do not drink, jump to B1.1

A10.1 if you have quit drinking, how long have you quit smoking? (months)

A10.2 2 How many years have you been drinking? (years)

A10.3 How many do you drinking currently/ before you quit drinking?

**PART B SOCIAL CAPITAL STATUS**

**B1 Social Participation**

B1.1 In the past 12 months, how often would you participate in formal groups (party or democratic parties’ elections, etc.)?

① Never ②Seldom ③Usually ④Often ⑤More Often

B1.2 In the past 12 months, how often would you participate in informal groups (square dance, interest clubs, etc.)?

① Never ②Seldom ③Usually ④Often ⑤More Often

B1.3 In the past 12 months, how often would you be community volunteer (coordinator, corridor manager, etc.)?

① Never ②Seldom ③Usually ④Often ⑤More Often

B1.4 In the past 12 months, how often would you take part in community services (health lecture, cultural activities etc.)?

① Never ②Seldom ③Usually ④Often ⑤More Often

**B2 Social Support**

B2.1 When you are in trouble, is there someone that provides you with mental support (i.e., comfort you)?

① Never ②Seldom ③Usually ④Often ⑤More Often

B2.2 When you are in trouble, is there someone that provides you with material support (i.e., lend you money)?

① Never ②Seldom ③Usually ④Often ⑤More Often

B2.3 When you are in trouble, are there any formal or informal groups that provide you with mental support (i.e., comfort you)?

① Never ②Seldom ③Usually ④Often ⑤More Often

B2.4 When you are in trouble, are there any formal or informal groups that provide you with material support (i.e., lend you money)?

① Never ②Seldom ③Usually ④Often ⑤More Often

**B3 Social Connection**

B3.1 How often do you contact with your children?

① Never ②Seldom ③Usually ④Often ⑤More Often

B3.2 How often do you contact with your relatives?

① Never ②Seldom ③Usually ④Often ⑤More Often

B3.3 How often do you contact with your friends/ neighbors?

① Never ②Seldom ③Usually ④Often ⑤More Often

**B4 Trust**

B4.1 Do you trust in your family members?

① Never ②Seldom ③Usually ④Often ⑤More Often

B4.2 Do you trust in your friends?

① Never ②Seldom ③Usually ④Often ⑤More Often

B4.3 Do you trust in someone who lives within one community/ village?

① Never ②Seldom ③Usually ④Often ⑤More Often

**B5 Cohesion**

B5.1 Do you care about what happened in your community/village?

① Never ②Seldom ③Usually ④Often ⑤More Often

B5.2 Do you think the community/village is more harmonious?

① Never ②Seldom ③Usually ④Often ⑤More Often

B5.3 Do you like the community/village you live now?

① Never ②Seldom ③Usually ④Often ⑤More Often

B5.4 You have a feeling of being in the community/village?

① Never ②Seldom ③Usually ④Often ⑤More Often

B5.5 Do you feel reluctant, if you have to move away from the community lived now?

① Never ②Seldom ③Usually ④Often ⑤More Often

**B6 Reciprocity**

B6.1 When your relatives are in trouble, will you provide help to them?

① Never ②Seldom ③Usually ④Often ⑤More Often

B6.2 When your friends/ neighbors are in trouble, will you provide help to them?

① Never ②Seldom ③Usually ④Often ⑤More Often

B6.3 When some strangers are in trouble, will you provide help to them?

① Never ②Seldom ③Usually ④Often ⑤More Often

**E. Self-Rated Depression Status**

E1. I have a good mood, whenever I get some trouble.

① Seldom ②Usually ③Often ④More Often

E2. I am in a good mood, not easy to get angry.

① Seldom ②Usually ③Often ④More Often

E3. I feel the best in the morning.

① Seldom ②Usually ③Often ④More Often

E4. I sleep very well at night.

① Seldom ②Usually ③Often ④More Often

E5. I eat as much as usual; my appetite is good.

① Seldom ②Usually ③Often ④More Often

E6. My weight has not changed

① Seldom ②Usually ③Often ④More Often

E7. My heartbeat is the same as usual.

① Seldom ②Usually ③Often ④More Often

E8. I won't feel tired for no reason.

① Seldom ②Usually ③Often ④More Often

E9. My mind is as clear as usual.

① Seldom ②Usually ③Often ④More Often

E10. I don't think it is difficult to do things as usual.

① Seldom ②Usually ③Often ④More Often

E11. I rarely feel annoyed in my heart.

① Seldom ②Usually ③Often ④More Often

E12. I have hope for the future

① Seldom ②Usually ③Often ④More Often

E13. I think it is easy to make decisions.

① Seldom ②Usually ③Often ④More Often

E14. I feel that I am a useful person, someone needs me.

① Seldom ②Usually ③Often ④More Often

E15. I have a very interesting life.

① Seldom ②Usually ③Often ④More Often

E16. I am still interested in things that are usually of interest.

① Seldom ②Usually ③Often ④More Often
