# Supplementary material for: Association between social capital and depression among older people: evidence from Anhui Province, China
Source: BMC Public Health. 2020 Oct 16;20:1560. doi: 10.1186/s12889-020-09657-7 (PMC7565750; doi:10.1186/s12889-020-09657-7)
Supplement: Supplementary file 2 — Additional file 2. Results of collinearity analysis. [file 12889_2020_9657_MOESM2_ESM.docx]

**Table S1** Results of collinearity analysis

|  | | | | | | | | |
| --- | --- | --- | --- | --- | --- | --- | --- | --- |
| Model | | Unstandardized Coefficients | | Standardized Coefficients | t | Sig. | Collinearity Statistics | |
|  |  | B | Std. Error | Beta |  |  | Tolerance | VIF |
| 1 | (Constant) | 18.345 | 3.966 |  | 4.626 | .000 |  |  |
|  | age | -.039 | .032 | -.026 | -1.225 | .221 | .856 | 1.169 |
|  | gender | -2.439 | .564 | -.107 | -4.328 | .000 | .645 | 1.552 |
|  | BMI | .036 | .067 | .011 | .540 | .589 | .933 | 1.072 |
|  | residence | -.189 | .492 | -.008 | -.384 | .701 | .839 | 1.191 |
|  | living status | .285 | .796 | .009 | .358 | .721 | .679 | 1.472 |
|  | marital status | .164 | .343 | .012 | .478 | .633 | .629 | 1.589 |
|  | education | .863 | .314 | .061 | 2.748 | .006 | .789 | 1.268 |
|  | smoking status | 1.181 | .499 | .058 | 2.365 | .018 | .651 | 1.537 |
|  | drinking status | .234 | .520 | .010 | .450 | .652 | .746 | 1.340 |
|  | social participation | .357 | .067 | .120 | 5.323 | .000 | .770 | 1.299 |
|  | social support | .163 | .054 | .076 | 3.042 | .002 | .622 | 1.608 |
|  | social connection | .782 | .120 | .173 | 6.513 | .000 | .557 | 1.797 |
|  | trust | .607 | .153 | .124 | 3.981 | .000 | .405 | 2.468 |
|  | cohesion | .318 | .069 | .133 | 4.584 | .000 | .465 | 2.152 |
|  | reciprocity | .301 | .094 | .090 | 3.214 | .001 | .503 | 1.986 |
| a. Dependent Variable: self-rated depression | | | | | | | | |
